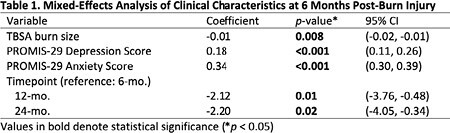# 70 Anxiety and Depression Increase Sleep Disturbances for Burn Survivors: A Burn Model System Study

**DOI:** 10.1093/jbcr/irae036.062

**Published:** 2024-04-17

**Authors:** Anika Kim, Kara McMullen, Barclay T Stewart, Jeffrey C Schneider, Colleen M Ryan, Paul Won, Haig A Yenikomshian

**Affiliations:** Keck School of Medicine at University of Southern California, Los Angeles, CA; University of Washington, Seattle, WA; Spaulding Rehabilitation Hospital/Harvard Medical School, Boston, MA; Massachusetts General Hospital/Shriners Children's, Boston, MA; Keck School of Medicine, Los Angeles, CA; University of Southern California, Los Angeles, CA; Keck School of Medicine at University of Southern California, Los Angeles, CA; University of Washington, Seattle, WA; Spaulding Rehabilitation Hospital/Harvard Medical School, Boston, MA; Massachusetts General Hospital/Shriners Children's, Boston, MA; Keck School of Medicine, Los Angeles, CA; University of Southern California, Los Angeles, CA; Keck School of Medicine at University of Southern California, Los Angeles, CA; University of Washington, Seattle, WA; Spaulding Rehabilitation Hospital/Harvard Medical School, Boston, MA; Massachusetts General Hospital/Shriners Children's, Boston, MA; Keck School of Medicine, Los Angeles, CA; University of Southern California, Los Angeles, CA; Keck School of Medicine at University of Southern California, Los Angeles, CA; University of Washington, Seattle, WA; Spaulding Rehabilitation Hospital/Harvard Medical School, Boston, MA; Massachusetts General Hospital/Shriners Children's, Boston, MA; Keck School of Medicine, Los Angeles, CA; University of Southern California, Los Angeles, CA; Keck School of Medicine at University of Southern California, Los Angeles, CA; University of Washington, Seattle, WA; Spaulding Rehabilitation Hospital/Harvard Medical School, Boston, MA; Massachusetts General Hospital/Shriners Children's, Boston, MA; Keck School of Medicine, Los Angeles, CA; University of Southern California, Los Angeles, CA; Keck School of Medicine at University of Southern California, Los Angeles, CA; University of Washington, Seattle, WA; Spaulding Rehabilitation Hospital/Harvard Medical School, Boston, MA; Massachusetts General Hospital/Shriners Children's, Boston, MA; Keck School of Medicine, Los Angeles, CA; University of Southern California, Los Angeles, CA; Keck School of Medicine at University of Southern California, Los Angeles, CA; University of Washington, Seattle, WA; Spaulding Rehabilitation Hospital/Harvard Medical School, Boston, MA; Massachusetts General Hospital/Shriners Children's, Boston, MA; Keck School of Medicine, Los Angeles, CA; University of Southern California, Los Angeles, CA

## Abstract

**Introduction:**

In aftercare, burn survivors have many challenges arising from their injuries. Sleep disturbance, despite its significant impact on quality of life, remains a poorly understood and studied consequence. This study investigates the interplay between sleep quality, mental health, and recovery in adult burn survivors.

**Methods:**

This retrospective study used the Burn Model System National Longitudinal Database (2015 to 2023) to examine sleep disturbance (SD) in adult burn patients. Subjects were divided into two groups based on their PROMIS-29 SD scores at 6, 12, 24 months, and 5 years follow-up: no to mild and moderate to severe SD. Mixed-effects modeling was used to compare the clinical characteristics of the two groups at 6 months. Person-level and site-level random effects were included to account for variations among subjects and treatment sites.

**Results:**

The study included 558 adult burn survivors with a mean age of 47.7 years and a mean Total Body Surface Area (TBSA) burn size of 16.0%. Mean SD scores were highest at 6 months (51.1) and decreased by 24 months (49.1) (higher scores = more SD). The study identified a positive association between SD and depression and anxiety scores (higher scores = more anxiety and depression). A one-unit increase in SD corresponded to a 0.184 increase in depression scores and a 0.343 increase in anxiety scores at 6 months (p-value < 0.001) (see Table 1). The association remained significant when controlling for person and burn injury site differences. No other significant differences were found between demographic or clinical characteristics at 6 months.

**Conclusions:**

This study highlights the complex interaction between SD, mental health, and recovery after burn injuries. Our data reveal that while subjects with depression and anxiety are more likely to experience SD after burn injury, there's a promising trend of improvement over time. These findings lay the foundation for improving targeted interventions and education in the clinical care of adult burn survivors.

**Applicability of Research to Practice:**

Insights from the study identify a subgroup of burn survivors who might benefit from personalized interventions addressing sleep, anxiety, and depression. Furthermore, integrating the expected sleep patterns after burn injuries into patient and caregiver education at discharge and providing patient-specific coping strategies will further enhance the recovery outlook and personalize the aftercare experience.